# *Thalassoglobus polymorphus* sp. nov., a novel Planctomycete isolated close to a public beach of Mallorca Island

**DOI:** 10.1007/s10482-020-01437-y

**Published:** 2020-06-24

**Authors:** Elena Rivas-Marin, Sandra Wiegand, Nicolai Kallscheuer, Mareike Jogler, Stijn H. Peeters, Anja Heuer, Mike S. M. Jetten, Christian Boedeker, Manfred Rohde, Damien P. Devos, Christian Jogler

**Affiliations:** 1 Centro Andaluz de Biología del Desarrollo, CSIC, Universidad Pablo de Olavide, Seville, Spain; 2 Institute for Biological Interfaces 5, Karlsruhe Institute of Technology, Eggenstein-Leopoldshafen, Germany; 3 Department of Microbiology, Radboud Universiteit, Nijmegen, The Netherlands; 4 Department of Microbial Interactions, Friedrich-Schiller University, Jena, Germany; 5 Leibniz Institute DSMZ, Brunswick, Germany; 6 Central Facility for Microscopy, Helmholtz Centre for Infection Research, Brunswick, Germany

**Keywords:** Marine bacteria, PVC superphylum, Mallorca coast, *Planctomicrobium piriforme*, *Thalassoglobus neptunius*

## Abstract

Access to axenic cultures is crucial to extend the knowledge of the biology, lifestyle or metabolic capabilities of bacteria from different phyla. The phylum *Planctomycetes* is an excellent example since its members display an unusual cell biology and complex lifestyles. As a contribution to the current collection of axenic planctomycete cultures, here we describe strain Mal48^T^ isolated from phytoplankton material sampled at the coast of S’Arenal close to Palma de Mallorca (Spain). The isolated strain shows optimal growth at pH 7.0–7.5 and 30 °C and exhibits typical features of Planctomycetes. Cells of the strain are spherical to pear-shaped, divide by polar budding with daughter cells showing the same shape as the mother cell, tend to aggregate, display a stalk and produce matrix or fimbriae. Strain Mal48^T^ showed 95.8% 16S rRNA gene sequence similarity with the recently described *Thalassoglobus neptunius* KOR42^T^. The genome sequence of the novel isolate has a size of 6,357,355 bp with a G+C content of 50.3%. A total of 4874 protein-coding genes, 41 tRNA genes and 2 copies of the 16S rRNA gene are encoded in the genome. Based on phylogenetic, morphological and physiological analyses, we conclude that strain Mal48^T^ (= DSM 100737^T^ = LMG 29019^T^) should be classified as the type strain of a new species in the genus *Thalassoglobus*, for which the name *Thalassoglobus polymorphus* sp. nov. is proposed.

## Introduction

The phylum *Planctomycetes* forms the medically, environmentally and biotechnologically important PVC superphylum together with *Verrucomicrobia*, *Lentisphaerae*, *Kirimatiellaeota* and *Chlamydiae* (Wagner and Horn 2006; Devos and Ward 2014; Wiegand et al. 2018). Planctomycetes are ubiquitous bacteria, which colonise a variety of environments from terrestrial to aquatic, marine or freshwater, in which they act as important contributors to the activity of the global carbon and nitrogen cycle (Wiegand et al. 2020). One example of such an activity includes members of the class *Candidatus* Brocadiae capable of performing anaerobic ammonium oxidation (anammox) (Strous et al. 1999). The anammox process is industrially exploited for removal of ammonia during wastewater treatment (Peeters and van Niftrik 2019). Members of the classes *Phycisphaerae* and *Planctomycetia* are frequently found attached to algal surfaces. Both, cultivation-dependent and -independent methods, have proven the frequent association of Planctomycetes with macroalgae (Bengtsson and Øvreås 2010; Bondoso et al. 2014, 2017; Lage and Bondoso 2014). The ability to attach to surfaces is prerequisite for biofilm formation on biotic and abiotic surfaces (Bengtsson and Øvreås 2010; Kohn et al. 2020a, b). Furthermore, the genomes of Planctomycetes code for enzymes putatively involved in the degradation of complex carbon substrates (Wecker et al. 2009; Wegner et al. 2013). This might be a decisive advantage in competitive environments since such compounds are one of the few sources of carbon and energy in the otherwise oligotrophic seawater (Lachnit et al. 2013; Jeske et al. 2013; Kim et al. 2016).

Historically, Planctomycetes were thought to display a number of exceptional traits. Amongst others, a compartmentalised cell plan, a nucleus-like structure and the lack of peptidoglycan were proposed (König et al. 1984; Fuerst and Webb 1991; Lindsay et al. 1997; Lonhienne et al. 2010). Due to the emergence of more sophisticated microscopic techniques and genetic tools allowing genetic modification of Planctomycetes, these misinterpretations have been resolved (Jogler et al. 2011; Rivas-Marín et al. 2016b). Peptidoglycan has been detected in several members of the phylum (Jeske et al. 2015; van Teeseling et al. 2015) and their internal compartments were found to be invaginations of the cytoplasmic membrane (Santarella-Mellwig et al. 2013; Acehan et al. 2014; Boedeker et al. 2017), with the exception of the anammoxosome of members of the class *Candidatus* Brocadiae (Niftrik et al. 2006). It is now well accepted that *Planctomycetes* is a peculiar phylum of bacteria featuring a diderm bacterial cell envelope architecture. Some strains have been shown to have expanded cytoplasmic membranes, a different composition of peptidoglycan or condensed DNA, but all of these characteristics are variations rather than exceptions to the Gram-negative cell plan (Devos 2014a, b; Boedeker et al. 2017; Rivas-Marín and Devos 2018).

Although most of the controversies have been resolved, the prospect of deciphering of the molecular and cellular biology of Planctomycetes is still very exciting. Several of their peculiarities need to be studied in depth. This is e.g. the case for their unusual mechanism of proliferation. Members of the class *Phycisphaerae* typically divide by binary fission, while species within the class *Planctomycetia* divide by budding. It has been also reported that related members of the proposed phylum ‘*Saltotorellota*’ are capable of switching between both mechanisms (Wiegand et al. 2019). Surprisingly, all Planctomycetes lack the canonical divisome protein FtsZ as well as some other ‘essential’ division proteins (Pilhofer et al. 2008; Rivas-Marín et al. 2016a; Rivas-Marin et al. 2020). Their complex endomembrane systems and their uncommon capacity to take up macromolecules are also subject of current studies (Boedeker et al. 2017). Planctomycetes appear to be resistant to many antibiotics (Cayrou et al. 2010; Godinho et al. 2019) and their estimated capacity to produce secondary metabolites is quite high (Jeske et al. 2013, 2016). This assumption is based on the presence of secondary metabolite-related gene clusters and the activity of small molecules produced and experimentally tested (Calisto et al. 2019; Graça et al. 2016; Jeske et al. 2016; Panter et al. 2019; Wiegand et al. 2020).

In this study, we describe a novel strain, Mal48^T^, which was isolated from phytoplankton sampled in the Mediterranean Sea close to Palma de Mallorca (Spain). Based on the results obtained, we conclude that strain Mal48^T^ represents a novel species of the recently described genus *Thalassoglobus* within the family *Planctomycetaceae* (Kohn et al. 2020a).

## Materials and methods

*Cultivation conditions and isolation* Strain Mal48^T^ was isolated on the 23th of September 2014 from phytoplankton collected at the coast of S’Arenal close to Palma de Mallorca (Spain) (sampling location: 39.5126 N 2.7470 E). After centrifugation of the sampling material, the pellet was resuspended in 100 µL sterile artificial seawater (ASW) and streaked on a plate containing M1H medium with *N*-acetylglucosamine (NAG) and ASW (designated M1H NAG ASW) (Kallscheuer et al. 2019a) solidified with 15 g/L agar and additionally supplemented with 200 mg/L ampicillin, 500 mg/L streptomycin and 20 mg/L cycloheximide. Plates were incubated at 28 °C for 3–4 weeks and isolated colonies were then streaked on fresh M1H NAG ASW plates. Initial amplification and sequencing of the 16S rRNA gene was performed as previously described (Rast et al. 2017). This step was included to ensure that the isolated strain is indeed a member of the phylum *Planctomycetes*.

*Physiological analyses* For temperature and pH optima determination M1H NAG ASW medium was used. The strain was cultivated at pH 8 at different temperatures ranging from 10 to 40 °C. For pH optimum identification 100 mM 2-(*N*-morpholino)ethanesulfonic acid (MES, pH 5.0–6.5), HEPES (pH 7.0–8.0), HEPPS (pH 8.5) or *N*-cyclohexyl-2-aminoethanesulfonic acid (CHES, pH 9.0–9.5) were used as buffering agents. Cultivations for determination of the pH optimum were performed at 28 °C. Growth was determined from optical density measurements at 600 nm (OD_600_) of triplicate cultures.

*Genome analysis* The genome of strain Mal48^T^ is available from NCBI under GenBank accession number CP036267 and the 16S rRNA gene sequence under accession number MK625061. Sequencing of the genome is described in a previous study (Wiegand et al. 2020). The primary metabolism was analysed by examining locally computed InterProScan (Mitchell et al. 2019) results cross-referenced with information from the UniProt database (UniProt 2019) and BlastP results of ‘typical’ protein sequences.

*Light microscopy and scanning electron microscopy* Phase contrast light microscopy and scanning electron microscopy were performed as previously described (Kallscheuer et al. 2019a).

*Phylogenetic analyses* The 16S rRNA gene-based phylogenetic analysis of strain Mal48^T^ was computed along with sequences of all described planctomycetal species (assessed in January 2020), including recently published isolates (Kohn et al. 2016, 2020a, b; Kulichevskaya et al. 2015; Boersma et al. 2019; Kallscheuer et al. 2019a; Dedysh et al. 2020). SINA was used to perform the 16S rRNA gene sequence alignment (Pruesse et al. 2012). The phylogenetic inference was performed employing a maximum likelihood approach with 1000 bootstraps, the nucleotide substitution model GTR, gamma distribution and estimation of proportion of invariable sites (GTRGAMMAI option) (Stamatakis 2014). Three 16S rRNA genes from members of the PVC superphylum, but outside of the phylum *Planctomycetes*, were used as outgroup (*Opitutus terrae*, acc. No. AJ229235; *Kiritimatiella glycovorans*, acc. no. NR_146840 and *Lentisphaera araneosa*, acc. no. NR_027571). The average nucleotide identity (ANI) was calculated using OrthoANI (Lee et al. 2016) and the average amino acid identity (AAI) was gained using the aai.rb script of the enveomics collection (Rodriguez-R and Konstantinidis 2016). The percentage of conserved proteins (POCP) was calculated as described (Qin et al. 2014). The *rpoB* gene sequences were extracted from the genome annotations and the sequence identities were determined as described (Bondoso et al. 2013) with Clustal Omega (Sievers et al. 2011). Alignment and matrix calculation were performed by extracting only those parts of the sequence that would have been sequenced with the described primer set. The unique single-copy core genome of all analysed genomes for the multi-locus sequence analysis (MLSA) was determined with Proteinortho5 (Lechner et al. 2011) (‘selfblast’ option enabled). The sequences of the obtained orthologous groups were aligned using MUSCLE v.3.8.31 (Edgar 2004). After clipping, partially aligned C- and N-terminal regions and poorly aligned internal regions were filtered using Gblocks (Castresana 2000). The final alignment of 709 ubiquitous genes with a combined length of 356,576 conserved amino acid residues was concatenated and clustered using the maximum likelihood method implemented by RaxML (Stamatakis 2014) (‘rapid bootstrap’ method and 500 bootstrap replicates).

## Results and discussion

### Phylogenetic inference

**Fig. 1:**
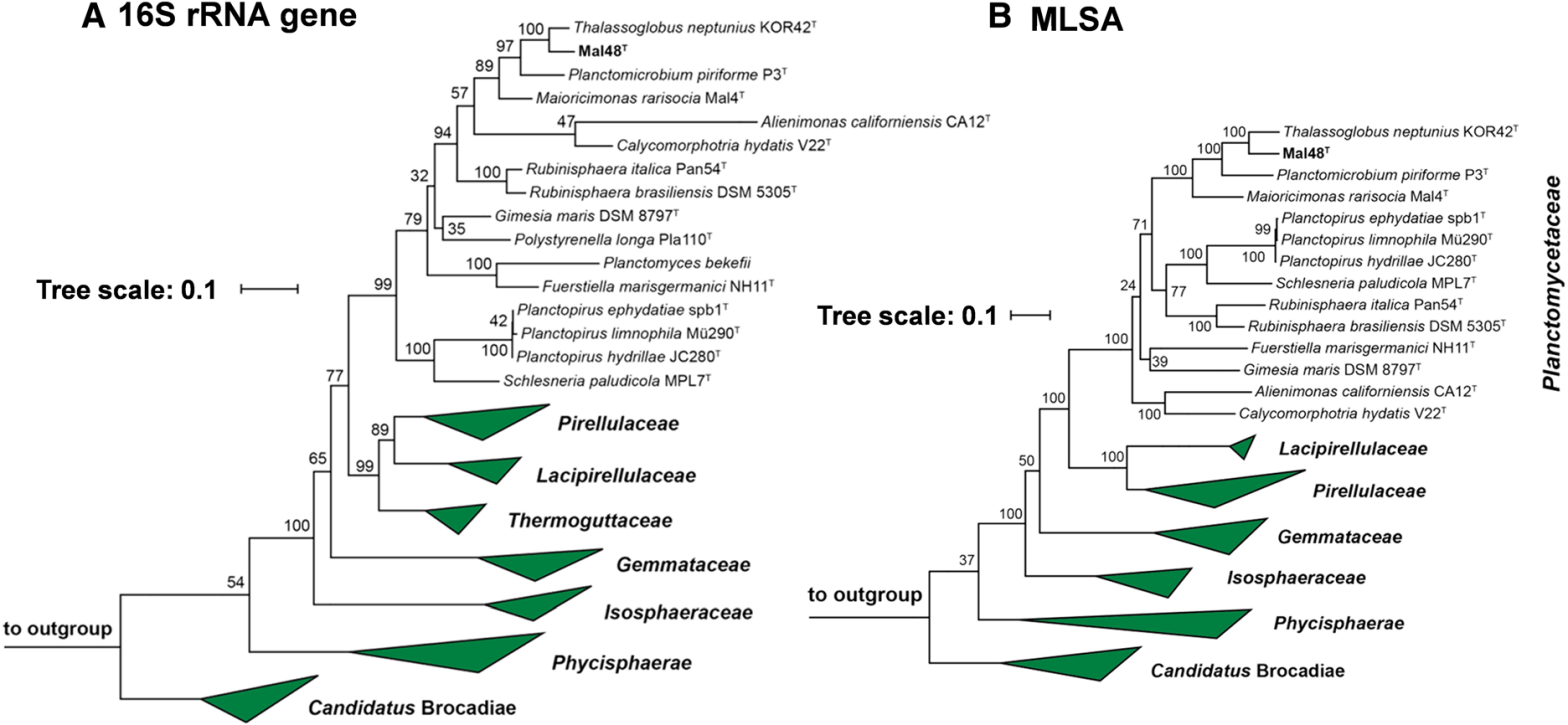
Maximum likelihood phylogenetic analysis showing the position of the novel strain Mal48^T^. 16S rRNA gene sequence (**a**)- and MLSA-based phylogeny (**b**) were computed as described in the “Materials and methods” section. Bootstrap values from 1000 re-samplings (500 re-samplings for MLSA) are given at the nodes (in  %)

**Fig. 2:**
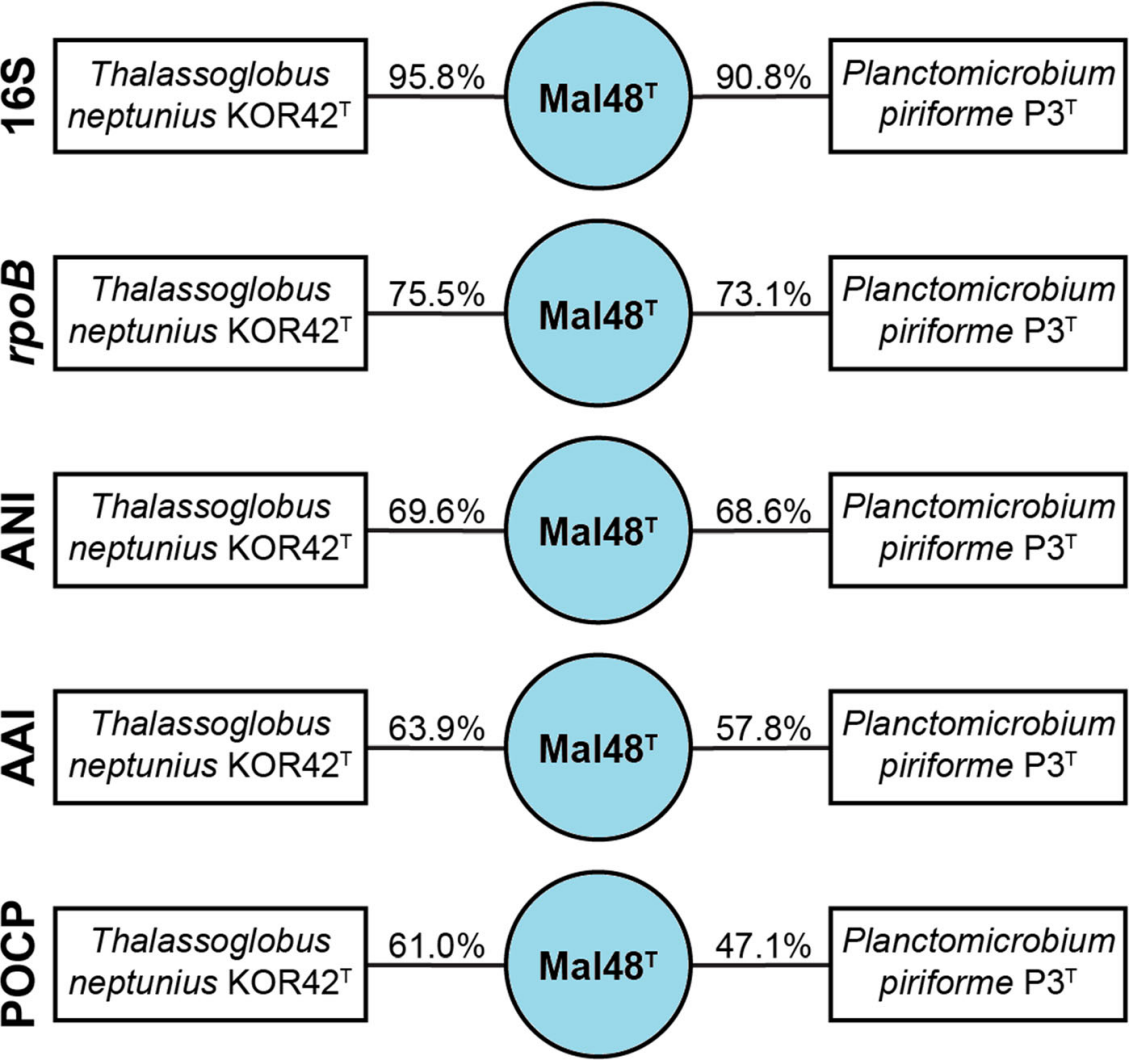
Comparison of phylogenetic markers for delineation of the novel isolate Mal48^T^. Methods used: 16S rRNA gene sequence identity (16S), *rpoB* gene identity (1200 bp fragment), average nucleotide identity (ANI), average amino acid identity (AAI) and percentage of conserved proteins (POCP)

During maximum likelihood phylogenetic analysis based on 16S rRNA gene sequences and MLSA, strain Mal48^T^ stably clustered with *Thalassoglobus neptunius* KOR42^T^, the type species of the recently described (but currently only effectively named) genus *Thalassoglobus* (Kohn et al. 2020a) (Fig. 1). The 16S rRNA gene sequence identity between Mal48^T^ and *T. neptunius* KOR42^T^ is 95.8% (Fig. 2). This value is above the proposed threshold for genera of 94.5% (Yarza et al. 2014) and thus strain Mal48^T^ likely represents a novel species within the genus *Thalassoglobus*. Accordingly, the 16S rRNA gene identity between Mal48^T^ and the next closest relative apart from KOR42^T^, *Planctomicrobium piriforme* P3^T^, is notably below this threshold, thereby confirming that strain Mal48^T^ is not a member of the genus *Planctomicrobium* (Fig. 2). For Planctomycetes, it has been found that 16S rRNA gene sequence similarity alone is not necessarily sufficient for delineation of species (Kohn et al. 2020b). Thus, phylogenetic assumptions on the genus level were further substantiated by reviewing the RNA polymerase β-subunit gene (*rpoB*) sequence identities (Bondoso et al. 2013), AAI (Konstantinidis and Tiedje 2005), POCP (Qin et al. 2014) or ANI (Lee et al. 2016). For *rpoB* gene identity, the threshold value for delineation of genera is defined by a range from 75.5 to 78% (Kallscheuer et al. 2019b). The *rpoB* identity of 75.5% between Mal48^T^ and *T. neptunius* KOR42^T^ reinforces placing both taxa in the same genus, but as separate species (species threshold: 96.3%). Comparison of the POCP between strain Mal48^T^ and KOR42^T^ yielded a value of 61.0%, which is above the proposed genus threshold of 50% (Qin et al. 2014). For AAI, the genus classification is defined to be between 60 and 80% (Luo et al. 2014). With an AAI of 63.9% placement of both strains in the same genus is not required, but can be justified. Finally, an ANI value of 69.9% for comparison of *T. neptunius* KOR42^T^ and strain Mal48^T^ confirms that both strains belong to separate species as the value is significantly below the species threshold of 95% (Kim et al. 2014).

Taken together, all the analysed phylogenetic markers (Fig. 2), as well as the phylogenetic trees (Fig. 1), support the conclusion that strain Mal48^T^ belongs to a novel species within the genus *Thalassoglobus.*

### Morphological and physiological analyses

**Fig. 3:**
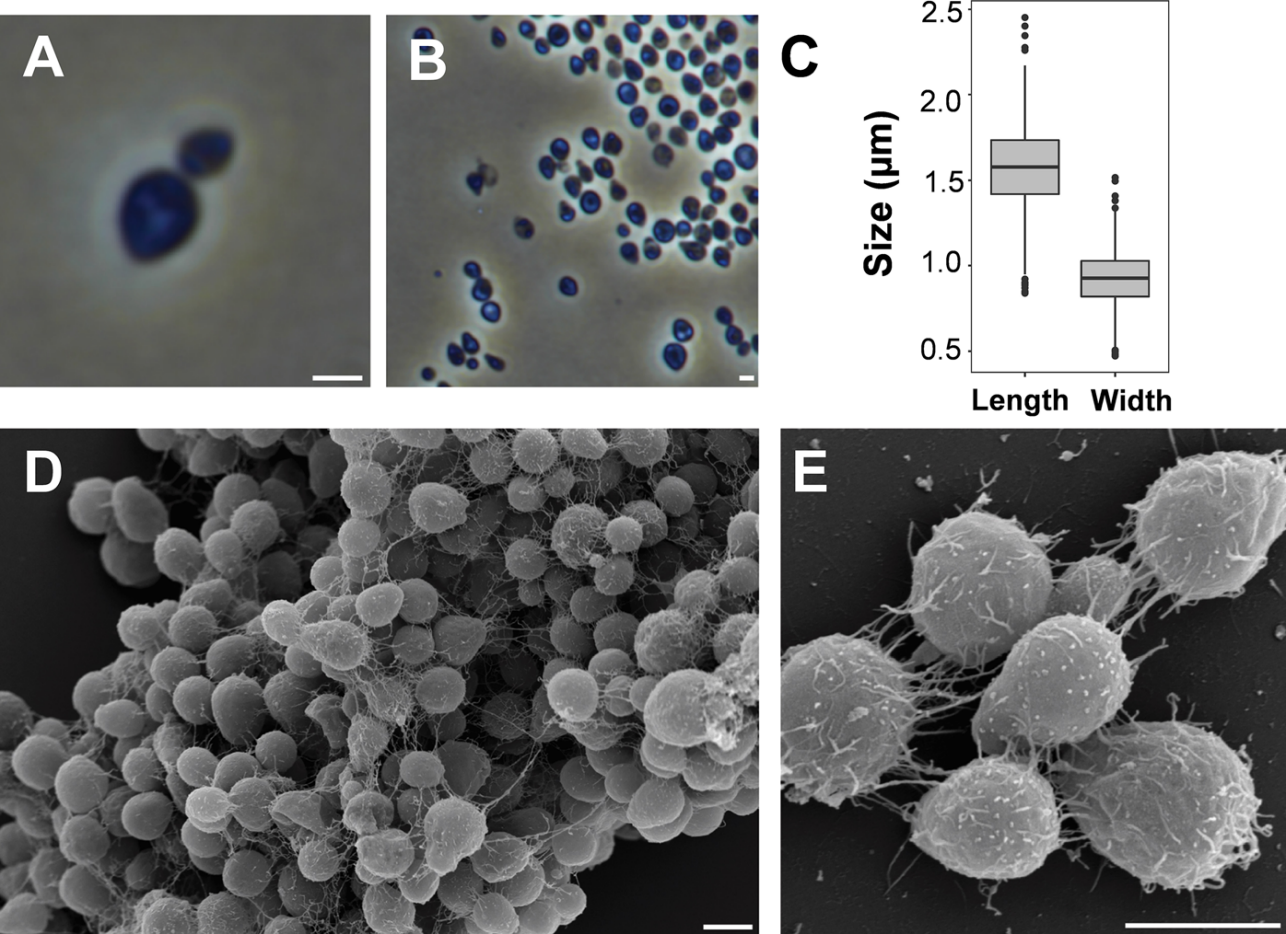
Morphology of strain Mal48^T^. The cell morphology was analysed by phase contrast (**a**, **b**) and scanning electron microscopy (**d**, **e**). Cells divide by budding (**a**) and produce dense aggregates (**d**). The scale bars are 1 µm. For determination of the cell size (**c**) at least 100 representative cells were counted manually or by using a semi-automated object count

The morphology of strain Mal48^T^ was characterised using phase contrast and scanning electron microscopy. Prior to the analysis, cells were harvested during the exponential growth phase from M1H NAG ASW medium. Detailed information about morphology and cell division is summarised in Table 1 in comparison to the current closest neighbours *T. neptunius* KOR42^T^ and *P. piriforme* P3^T^. Cells of strain Mal48^T^ are quite heterogeneous in shape; ranging from spherical to pear-shaped with different intermediate forms (Fig. 3b, d). The average cell size was determined to be 1.6 ± 0.3 µm × 0.9 ± 0.2 µm (Fig. 3c). Beige-coloured colonies were observed on solid medium, indicating a lack of carotenoid production. Mal48^T^ cells usually form aggregates (Fig. 3d) and divide by polar budding (Fig. 3a) with the daughter cells showing the same shape as the mother cell, as was also observed for *P. piriforme* P3^T^. The surface of the cells of strain Mal48^T^ is covered with matrix or fibre (Fig. 3d, e). Cells have a stalk; however, a holdfast structure was not observed during electron microscopic analysis. In contrast to the two strains used for comparison, crateriform structures could not be observed on the cell surface of strain Mal48^T^.

**Table 1:** Phenotypic and genotypic features of strain Mal48^T^ in comparison to *T. neptunius* KOR42^T^ and *P. piriforme* P3^T^

Characteristics	Mal48^T^	KOR42^T^*	P3^T^**
*Phenotypic features*			
Color	Beige	Cream	White
Size	1.6 × 0.9 µm	1.7 µm (diameter)	1.7–2.8 × 0.9–1.3 µm
Shape	Pear-shaped or spherical	Spherical	Ellipsoidal to pear-shaped
Motility	No	No	Yes
Temperature range (optimum) (° C)	15–36 (30)	22–36 (33)	10–30 (20–28)
pH range (optimum)	6.5–8.0 (7.5)	5.5–8.5 (7.0–7.5)	4.2–7.1 (6.0–6.5)
Aggregates	Yes	Yes	Yes
Division	Budding	Budding	Budding
Dimorphic life cycle	n.o.	n.o.	Yes
Flagella	n.o.	n.o.	Yes
Crateriform structures	n.o.	Yes	Yes, polar
Fimbriae	Yes, overall matrix or fiber	Few fibers	Yes
Capsule	n.o.	n.o.	n.d.
Bud shape	Like mother cell	Round	Like mother cell
Budding pole	Polar	n.o.	Polar
Stalk	Yes	n.o.	Yes
Holdfast structure	n.o.	Yes	Yes
*Genomic features*			
Genome size (bp)	6,357,355	6,734,412	6,317,004
Plasmids (bp)	n.o.	n.o.	n.d.
G+C content (%)	50.3	52.8	58.8
Completeness (%)	97.41	96.55	95.69
Contamination (%)	0	0	1.72
Protein-coding genes	4874	5508	5050
Protein-coding genes/Mb	767	818	799
Hypothetical proteins	1987	2516	2814
Coding density (%)	84.9	85.7	85.8
16S rRNA genes	2	1	1
tRNA genes	41	70	53

*Genomic data from GenBank Acc. No. SIHI00000000

**Genomic data from GenBank Acc. No. NZ_FOQD00000000

*n.o.* not observed, *n.d.* not determined

**Fig. 4:**
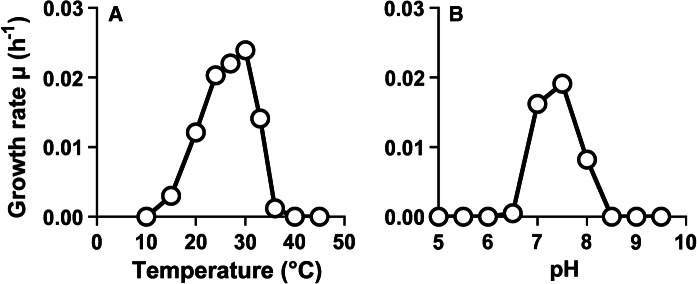
Temperature and pH optimum of strain Mal48^T^. Cultivations at different temperatures (**a**) were performed at pH 8.0. Cultivations at different pH values (**b**) were conducted at 28 °C. The growth rates were obtained from the slope of the plot of ln(OD_600_) against the cultivation time for each tested condition. Data from triplicate cultivations was used

In physiological analyses, strain Mal48^T^ was found to preferentially grow at 30 °C and pH 7.5, however, cells were able to proliferate over a range of 15–36 °C and pH 6.5–8.0 (Fig. 4). The maximal growth rate in M1H NAG ASW medium was found to be 0.024 h^−1^, which corresponds to a generation time of approximately 29 h. Optimal conditions regarding temperature and pH are only slightly different between Mal48^T^ and *T. neptunius* KOR42^T^. In contrast, *P. piriforme* P3^T^ prefers considerably lower temperatures and moderate acidic conditions. These conditions likely reflect the natural conditions in which the strain was isolated (littoral wetland of a boreal lake).

### Genomic characteristics

A comparison of the genomic characteristics of strain Mal48^T^, *T. neptunius* KOR42^T^ and *P. piriforme* P3^T^ is outlined in Table 1. The genome of strain Mal48^T^ has a size of 6.4 Mb, which is in the same range as in *T. neptunius* KOR42^T^ (6.7 Mb) and *P. piriforme* P3^T^ (6.3 Mb), however, the G+C content is slightly lower (50.3% for Mal48^T^, 52.8% for KOR42^T^, 58.8% for P3^T^). 4874 putative protein-encoding genes were identified by automated gene prediction and annotation, of which 40.8% (1987 genes) encode hypothetical proteins. These values correspond to 767 protein-coding genes per Mb, yielding a coding density of 84.9%. This parameter is in the same range also in the other species. Similar to the close relatives chosen for comparison, strain Mal48^T^ lacks plasmids. The number of tRNA genes is lower compared to *T. neptunius* KOR42^T^ and *P. piriforme* P3^T^. Strain Mal48^T^ harbours two copies of the 16S rRNA gene, whereas a single gene was found in *T. neptunius* KOR42^T^ and *P. piriforme* P3^T^.

### Genome-encoded features of the primary carbon metabolism

In order to check for the presence of key metabolic enzymes participating in the central carbon metabolism, we performed a genome-based analysis of strain Mal48^T^ in comparison to *T. neptunius* KOR42^T^ and *P. piriforme* P3^T^ (Table 2). Glycolysis, pentose phosphate pathway, gluconeogenesis and the tricarboxylic acid (TCA) cycle, including anaplerotic reactions, were included in the analysis. All three strains harbour genes coding for enzymes of the Embden-Meyerhof-Parnas (EMP) pathway (the most common glycolytic pathway) with a noticeable lack of the gene *pykF* encoding the pyruvate kinase I in the genome of strain Mal48^T^. This enzyme catalyses the conversion of phosphoenolpyruvate to pyruvate in a substrate-level phosphorylation reaction. A potential lack of this enzyme is likely compensated by the phosphotransferase system-dependent uptake of glucose, which uses phosphoenolpyruvate as phosphate donor to yield pyruvate and glucose-6-phosphate. This said, we assume that the glycolytic route is functional in strain Mal48^T^. All genes required for a functional TCA cycle were identified in all three strains. In addition to the EMP, two other sugar catabolic pathways are present in bacteria: the pentose phosphate pathway and the Entner-Doudoroff pathway. Strain Mal48^T^ possess all the genes required for the reactions of the pentose phosphate pathway. With regard to the Entner-Doudoroff pathway, candidate genes coding for a putative 2-dehydro-3-deoxyphosphogluconate aldolase and a phosphogluconate dehydratase were found.

**Table 2:** Genome-based primary metabolism of strain Mal48^T^ compared to the closest related species *T. neptunius* KOR42^T^ and *P. piriforme* P3^T^

Enzyme	EC number	Gene	Mal48^T^	KOR42^T^*	P3^T^**
*Glycolysis (Embden*–*Meyerhof*–*Parnas pathway)*					
Glucose-6-phosphate isomerase	5.3.1.9	*pgi*	Mal48_03960	Y	Y
ATP-dependent 6-phosphofructokinase isozyme 1	2.7.1.11	*pfkA*	Mal48_01100	Y	Y
Fructose-bisphosphate aldolase class 2	4.1.2.13	*fbaA*	Mal48_20830	Y	Y
Triosephosphate isomerase	5.3.1.1	*tpiA*	Mal48_48530	Y	Y
Glyceraldehyde-3-phosphate dehydrogenase	1.2.1.12	*gapA*	Mal48_05570	Y	Y
Phosphoglycerate kinase	2.7.2.3	*pgk*	Mal48_37300	Y	Y
2,3-bisphosphoglycerate-independent phosphoglycerate mutase	5.4.2.12	*gpmI*	Mal48_16500	Y	n.a.
2,3-bisphosphoglycerate-dependent phosphoglycerate mutase	5.4.2.11	*gpmA*	N	N	Y
Enolase	4.2.1.11	*eno*	Mal48_22830	Y	Y
Pyruvate kinase I	2.7.1.40	*pykF*	N	Y	Y
Pyruvate dehydrogenase complex	1.2.4.1/	*aceEF*	Mal48_00570/Mal48_18480	Y	Y
	2.3.1.12				
*Gluconeogenesis*					
Phosphoenolpyruvate synthase	2.7.9.2	*ppsA*	N	N	n.a.
Pyruvate, phosphate dikinase	2.7.9.1	*ppdK*	Mal48_37150	Y	Y
Pyruvate carboxylase.	6.4.1.1	*pyc*	Mal48_22860	Y	Y
Phosphoenolpyruvate carboxykinase (ATP)	4.1.1.49	*pckA*	N	N	Y
Phosphoenolpyruvate carboxykinase (GTP)	4.1.1.32	*pckG*	N	N	N
Phosphoenolpyruvate carboxykinase (diphosphate)	4.1.1.38	*PEPCK*	Mal48_16500	Y	n.a.
Fructose-1,6-bisphosphatase class 2	3.1.3.11	*glpX*	N	N	n.a.
Fructose-1,6-bisphosphatase class 1	3.1.3.11	*fbp*	N	Y	n.a.
Pyrophosphate–fructose 6-phosphate 1-phosphotransferase	2.7.1.90	*pfp*	Mal48_08170	Y	Y
*Pentose phosphate pathway*					
Glucose-6-phosphate 1-dehydrogenase	1.1.1.49	*zwf*	Mal48_19300	Y	Y
6-phosphogluconolactonase	3.1.1.31	*pgl*	Mal48_19400	Y	Y
			Mal48_19410		
			Mal48_41520		
			Mal48_38280		
6-phosphogluconate dehydrogenase, decarboxylating	1.1.1.44	*gndA*	Mal48_13910	Y	Y
Transketolase 2	2.2.1.1	*tktB*	Mal48_26790	Y	Y
Transaldolase B	2.2.1.2	*talB*	Mal48_06240	Y	Y
*KDPG (Entner*–*Doudoroff pathway)*					
KDPG aldolase	4.1.2.14	*eda*	Mal48_44770	Y	Y
Phosphogluconate dehydratase	4.2.1.12	*edd*	Mal48_25960	Y (candidate)	Y
			(candidate)		
*TCA cycle*					
Citrate synthase	2.3.3.16	*gltA*	Mal48_29080	Y	Y
Aconitate hydratase A	4.2.1.3	*acnA*	Mal48_48270	Y	Y
Isocitrate dehydrogenase [NADP]	1.1.1.42	*icd*	Mal48_24210	Y	Y
2-oxoglutarate dehydrogenase complex	1.2.4.2/	*sucAB*	Mal48_06600	Y	Y
	2.3.1.61		Mal48_24080		
Succinate-CoA ligase complex	6.2.1.5	*sucCD*	Mal48_27760/	Y	Y
			Mal48_48200		
Succinate dehydrogenase complex	1.3.5.1	*sdhABC*	Mal48_12710/Mal48_12720/	Y	Y
			Mal48_12700		
Fumarate hydratase class I, an/aerobic	4.2.1.2	*fumAB*	N	N	n.a.
Fumarate hydratase class II	4.2.1.2	*fumC*	Mal48_04830	Y	Y
Malate dehydrogenase	1.1.1.37	*mdh*	Mal48_29220	Y	Y
*Glyoxylate shunt*					
Isocitrate lyase	4.1.3.1	*aceA*	N	N	n.a.
Malate synthase G	2.3.3.9	*glcB*	N	N	n.a.

*Genomic data from GenBank Acc. No. SIHI00000000

**Genomic data from GenBank Acc. No. NZ_FOQD00000000. Presence of a gene in *T. neptunius* KOR42^T^ and *P. piriforme* P3^T^ is indicated by ‘Y’ and absence is indicated by ‘N’

*n.a.* not available

For de novo sugar biosynthesis (gluconeogenesis), the three strains only possess a few of the enzyme classes reported to participate in this anabolic pathway, however, this minimal set appears sufficient for a functional anabolic route.

All three strains lack the glyoxylate shunt, which is a shortened TCA cycle typically required for anaplerosis during growth on acetate. This route is typically found in bacteria capable of using acetate or fatty acids as sole carbon and energy source. Absence of the glyoxylate shunt suggests that the strains are either not capable of using such compounds as sole source of energy and carbon source or that they harbour alternative pathways for this purpose. Except for the lack of the glyoxlate shunt, all three species probably have a canonical primary carbon metabolism as found in most aerobic heterotrophic bacteria.

Taken together, our physiological, morphological, genomic and phylogenetic analyses led to the conclusion that strain Mal48^T^ (= DSM 100737^T^ = LMG 29019^T^) represents a novel species within the recently described genus *Thalassoglobus*, for which we propose the name *Thalassoglobus polymorphus* sp. nov., with strain Mal48^T^ as the type strain.

### *Thalassoglobus polymorphus* sp. nov.

*Thalassoglobus polymorphus* (po.ly.mor’phus. N.L. masc. adj. *polymorphus* (from Gr. masc. adj. *polymorphos*) multiform, polymorphic; corresponding to the varied shapes of the cells).

Cells are typically pear-shaped (1.6 × 0.9 µm), but can also have a roundish or ovoid shape. Cells produce matrix or fibre and tend to aggregate. Cells form beige colonies. Optimal temperature and pH for growth of the type strain are 30 °C and pH 7.5, respectively. Grows at 15–36 °C. The pH range for growth is narrow (pH 6.5–8.0); no growth is observed at pH 6.0 (or lower) and pH 8.5 (or higher). The genome of the type strain has a G+C content of 50.3%.

The type strain Mal48^T^ (= DSM 100737^T^ = LMG 29019^T^, deposited as Malle48) was isolated from phytoplankton collected in the Mediterranean Sea close to S’Arenal, Palma de Mallorca in September 2014. The genome sequence (accession number CP036267) and 16S rRNA gene sequence (accession number MK625061) of strain Mal48^T^ are available from GenBank.

## References

[CR1] Acehan D, Santarella-Mellwig R, Devos DP (2014). A bacterial tubulovesicular network. J Cell Sci.

[CR2] Bengtsson MM, Øvreås L (2010). Planctomycetes dominate biofilms on surfaces of the kelp *Laminaria hyperborea*. BMC Microbiol.

[CR3] Boedeker C, Schüler M, Reintjes G (2017). Determining the bacterial cell biology of Planctomycetes. Nat Commun.

[CR4] Boersma AS, Kallscheuer N, Wiegand S (2019). *Alienimonas californiensis* gen. nov. sp. nov., a novel Planctomycete isolated from the kelp forest in Monterey Bay. Antonie Van Leeuwenhoek.

[CR5] Bondoso J, Harder J, Lage OM (2013). *rpoB* gene as a novel molecular marker to infer phylogeny in *Planctomycetales*. Antonie Van Leeuwenhoek.

[CR6] Bondoso J, Balagué V, Gasol JM, Lage OM (2014). Community composition of the Planctomycetes associated with different macroalgae. FEMS Microbiol Ecol.

[CR7] Bondoso J, Godoy-Vitorino F, Balagué V (2017). Epiphytic Planctomycetes communities associated with three main groups of macroalgae. FEMS Microbiol Ecol.

[CR8] Calisto R, Sæbø EF, Storesund JE, Øvreås L, Herfindal L, Lage OM (2019). Anticancer activity in planctomycetes. Front Mar Sci.

[CR9] Castresana J (2000). Selection of conserved blocks from multiple alignments for their use in phylogenetic analysis. Mol Biol Evol.

[CR10] Cayrou C, Raoult D, Drancourt M (2010). Broad-spectrum antibiotic resistance of Planctomycetes organisms determined by Etest. J Antimicrob Chemother.

[CR11] Dedysh SN, Henke P, Ivanova AA (2020). 100-year-old enigma solved: identification, genomic characterization and biogeography of the yet uncultured *Planctomyces bekefii*. Environ Microbiol.

[CR12] Devos DP (2014). PVC bacteria: variation of, but not exception to, the Gram-negative cell plan. Trends Microbiol.

[CR13] Devos DP (2014). Re-interpretation of the evidence for the PVC cell plan supports a Gram-negative origin. Antonie Van Leeuwenhoek.

[CR14] Devos DP, Ward NL (2014). Mind the PVCs. Environ Microbiol.

[CR15] Edgar RC (2004). MUSCLE: multiple sequence alignment with high accuracy and high throughput. Nucleic Acids Res.

[CR16] Fuerst JA, Webb RI (1991). Membrane-bounded nucleoid in the eubacterium *Gemmata obscuriglobus*. Proc Natl Acad Sci USA.

[CR17] Godinho O, Calisto R, Øvreås L (2019). Antibiotic susceptibility of marine Planctomycetes. Antonie Van Leeuwenhoek.

[CR18] Graça AP, Calisto R, Lage OM (2016). Planctomycetes as novel source of bioactive molecules. Front Microbiol.

[CR19] Jeske O, Jogler M, Petersen J (2013). From genome mining to phenotypic microarrays: Planctomycetes as source for novel bioactive molecules. Antonie Van Leeuwenhoek.

[CR20] Jeske O, Schüler M, Schumann P (2015). Planctomycetes do possess a peptidoglycan cell wall. Nat Commun.

[CR21] Jeske O, Surup F, Ketteniß M (2016). Developing techniques for the utilization of planctomycetes as producers of bioactive molecules. Front Microbiol.

[CR22] Jogler C, Glöckner FO, Kolter R (2011). Characterization of *Planctomyces limnophilus* and development of genetic tools for its manipulation establish it as a model species for the phylum Planctomycetes. Appl Environ Microbiol.

[CR23] Kallscheuer N, Jogler M, Wiegand S (2019). *Rubinisphaera italica* sp. nov. isolated from a hydrothermal area in the Tyrrhenian Sea close to the volcanic island Panarea. Antonie Van Leeuwenhoek.

[CR24] Kallscheuer N, Wiegand S, Peeters SH (2019). Description of three bacterial strains belonging to the new genus *Novipirellula* gen. nov., reclassificiation of *Rhodopirellula rosea* and *Rhodopirellula caenicola* and readjustment of the genus threshold of the phylogenetic marker *rpoB* for *Planctomycetaceae*. Antonie Van Leeuwenhoek.

[CR25] Kim M, Oh HS, Park SC, Chun J (2014). Towards a taxonomic coherence between average nucleotide identity and 16S rRNA gene sequence similarity for species demarcation of prokaryotes. Int J Syst Evol Microbiol.

[CR26] Kim JW, Brawley SH, Prochnik S (2016). Genome Analysis of Planctomycetes Inhabiting Blades of the Red Alga *Porphyra umbilicalis*. PLoS ONE.

[CR27] Kohn T, Heuer A, Jogler M (2016). *Fuerstia marisgermanicae* gen. nov., sp. nov., an Unusual Member of the Phylum Planctomycetes from the German Wadden Sea. Front Microbiol.

[CR28] Kohn T, Rast P, Wiegand S (2020). The microbiome of *Posidonia oceanica* seagrass leaves can be dominated by Planctomycetes. Front Microbiol.

[CR29] Kohn T, Wiegand S, Boedeker C (2020). *Planctopirus ephydatiae*, a novel Planctomycete isolated from a freshwater sponge. Syst Appl Microbiol.

[CR30] König E, Schlesner H, Hirsch P (1984). Cell wall studies on budding bacteria of the *Planctomyces/Pasteuria* group and on a *Prosthecomicrobium* sp. Arch Microbiol.

[CR31] Konstantinidis KT, Tiedje JM (2005). Genomic insights that advance the species definition for prokaryotes. Proc Natl Acad Sci USA.

[CR32] Kulichevskaya IS, Ivanova AA, Detkova EN (2015). *Planctomicrobium piriforme* gen. nov., sp. nov., a stalked planctomycete from a littoral wetland of a boreal lake. Int J Syst Evol Microbiol.

[CR33] Lachnit T, Fischer M, Künzel S (2013). Compounds associated with algal surfaces mediate epiphytic colonization of the marine macroalga *Fucus vesiculosus*. FEMS Microbiol Ecol.

[CR34] Lage OM, Bondoso J (2014). Planctomycetes and macroalgae, a striking association. Front Microbiol.

[CR35] Lechner M, Findeiß S, Steiner L (2011). Proteinortho: detection of (Co-)orthologs in large-scale analysis. BMC Bioinform.

[CR36] Lee I, Ouk Kim Y, Park S-C, Chun J (2016). OrthoANI: an improved algorithm and software for calculating average nucleotide identity. Int J Syst Evol Microbiol.

[CR37] Lindsay M, Webb R, Fuerst J (1997). Pirellulosomes: a new type of membrane-bounded cell compartment in Planctomycete bacteria of the genus *Pirellula*. Microbiology.

[CR38] Lonhienne TGA, Sagulenko E, Webb RI (2010). Endocytosis-like protein uptake in the bacterium *Gemmata obscuriglobus*. Proc Natl Acad Sci USA.

[CR39] Luo C, Rodriguez-R LM, Konstantinidis KT (2014). MyTaxa: an advanced taxonomic classifier for genomic and metagenomic sequences. Nucleic Acids Res.

[CR40] Mitchell AL, Attwood TK, Babbitt PC (2019). InterPro in 2019: improving coverage, classification and access to protein sequence annotations. Nucleic Acids Res.

[CR41] Niftrik LA, Fuerst JA, Damsté JSS (2006). The anammoxosome: an intracytoplasmic compartment in anammox bacteria. FEMS Microbiol Lett.

[CR42] Panter F, Garcia R, Thewes A (2019). Production of a dibrominated aromatic secondary metabolite by a planctomycete implies complex interaction with a macroalgal host. ACS Chem Biol.

[CR43] Peeters SH, van Niftrik L (2019). Trending topics and open questions in anaerobic ammonium oxidation. Curr Opin Chem Biol.

[CR44] Pilhofer M, Rappl K, Eckl C (2008). Characterization and evolution of cell division and cell wall synthesis genes in the bacterial phyla *Verrucomicrobia, Lentisphaerae, Chlamydiae*, and *Planctomycetes* and phylogenetic comparison with rRNA genes. J Bacteriol.

[CR45] Pruesse E, Peplies J, Glöckner FO (2012). SINA: accurate high-throughput multiple sequence alignment of ribosomal RNA genes. Bioinformatics.

[CR46] Qin Q-L, Xie B-B, Zhang X-Y (2014). A proposed genus boundary for the prokaryotes based on genomic insights. J Bacteriol.

[CR47] Rast P, Glöckner I, Boedeker C (2017). Three novel species with peptidoglycan cell walls form the new genus *Lacunisphaera* gen. nov. in the family *Opitutaceae* of the verrucomicrobial subdivision 4. Front Microbiol.

[CR48] Rivas-Marín E, Devos DP (2018). The Paradigms They Are a-Changin’: past, present and future of PVC bacteria research. Antonie Van Leeuwenhoek.

[CR49] Rivas-Marin E, Peeters SH, Claret Fernández L (2020). Non-essentiality of canonical cell division genes in the planctomycete *Planctopirus limnophila*. Sci Rep.

[CR50] Rivas-Marín E, Canosa I, Devos DP (2016). Evolutionary cell biology of division mode in the bacterial *Planctomycetes*-*Verrucomicrobia*-*Chlamydiae* superphylum. Front Microbiol.

[CR51] Rivas-Marín E, Canosa I, Santero E, Devos DP (2016). Development of genetic tools for the manipulation of the planctomycetes. Front Microbiol.

[CR52] Rodriguez-R LM, Konstantinidis KT (2016). The enveomics collection: a toolbox for specialized analyses of microbial genomes and metagenomes. PeerJ Preprints.

[CR53] Santarella-Mellwig R, Pruggnaller S, Roos N (2013). Three-dimensional reconstruction of bacteria with a complex endomembrane system. PLoS Biol.

[CR54] Sievers F, Wilm A, Dineen D (2011). Fast, scalable generation of high-quality protein multiple sequence alignments using Clustal Omega. Mol Syst Biol.

[CR55] Stamatakis A (2014). RAxML version 8: a tool for phylogenetic analysis and post-analysis of large phylogenies. Bioinformatics.

[CR56] Strous M, Fuerst JA, Kramer EH (1999). Missing lithotroph identified as new planctomycete. Nature.

[CR57] van Teeseling MCF, Mesman RJ, Kuru E (2015). Anammox Planctomycetes have a peptidoglycan cell wall. Nat Commun.

[CR58] Wagner M, Horn M (2006). The *Planctomycetes*, *Verrucomicrobia*, *Chlamydiae* and sister phyla comprise a superphylum with biotechnological and medical relevance. Curr Opin Biotechnol.

[CR59] Wecker P, Klockow C, Ellrott A (2009). Transcriptional response of the model planctomycete *Rhodopirellula baltica* SH1^T^ to changing environmental conditions. BMC Genom.

[CR60] Wegner CE, Richter-Heitmann T, Klindworth A (2013). Expression of sulfatases in *Rhodopirellula baltica* and the diversity of sulfatases in the genus *Rhodopirellula*. Mar Genomics.

[CR61] Wiegand S, Jogler M, Jogler C (2018). On the maverick Planctomycetes. FEMS Microbiol Rev.

[CR62] Wiegand S, Jogler M, Kohn T et al (2019) The novel shapeshifting bacterial phylum *Saltatorellota*. bioRxiv, 817700. 10.1101/817700

[CR63] Wiegand S, Jogler M, Boedeker C (2020). Cultivation and functional characterization of 79 planctomycetes uncovers their unique biology. Nat Microbiol.

[CR64] Yarza P, Yilmaz P, Pruesse E (2014). Uniting the classification of cultured and uncultured bacteria and archaea using 16S rRNA gene sequences. Nat Rev Microbiol.

